# Assessment of Stem Volume on Plots Using Terrestrial Laser Scanner: A Precision Forestry Application

**DOI:** 10.3390/s21010301

**Published:** 2021-01-05

**Authors:** Dimitrios Panagiotidis, Azadeh Abdollahnejad, Martin Slavík

**Affiliations:** Department of Forest Management, Faculty of Forestry and Wood Sciences, Czech University of Life Sciences (CZU Prague), Kamýcká 129, 165 21 Prague, Czech Republic; abdolahnejad@fld.czu.cz (A.A.); mslavik@fld.czu.cz (M.S.)

**Keywords:** forest biometrics, managed forest, 3D point cloud, stem-level assessment, circle fitting, hierarchical cluster analysis, modeling stem volume

## Abstract

Timber volume is an important asset, not only as an ecological component, but also as a key source of present and future revenues, which requires precise estimates. We used the Trimble TX8 survey-grade terrestrial laser scanner (TLS) to create a detailed 3D point cloud for extracting total tree height and diameter at breast height (1.3 m; DBH). We compared two different methods to accurately estimate total tree heights: the first method was based on a modified version of the local maxima algorithm for treetop detection, “H_TTD_”, and for the second method we used the centers of stem cross-sections at stump height (30 cm), “H_TSP_”. DBH was estimated by a computationally robust algebraic circle-fitting algorithm through hierarchical cluster analysis (HCA). This study aimed to assess the accuracy of these descriptors for evaluating total stem volume by comparing the results with the reference tree measurements. The difference between the estimated total stem volume from H_TTD_ and measured stems was 2.732 m^3^ for European oak and 2.971 m^3^ for Norway spruce; differences between the estimated volume from H_TSP_ and measured stems was 1.228 m^3^ and 2.006 m^3^ for European oak and Norway spruce, respectively. The coefficient of determination indicated a strong relationship between the measured and estimated total stem volumes from both height estimation methods with an R^2^ = 0.89 for H_TTD_ and R^2^ = 0.87 for H_TSP_ for European oak, and R^2^ = 0.98 for both H_TTD_ and H_TSP_ for Norway spruce. Our study has demonstrated the feasibility of finer-resolution remote sensing data for semi-automatic stem volumetric modeling of small-scale studies with high accuracy as a potential advancement in precision forestry.

## 1. Introduction

Forests are natural dynamic systems that are constantly responding to socio-environmental factors. Tree height and diameter are the most common descriptors for estimating basal area (BA), biomass, and stem volume [1,2,3,4]. However, stem form is another important consideration for volume estimation [5]. Tree shape varies due to differences in neighborhood species composition [6], genetic factors [7], climatic factors [8], silvicultural treatments, and forest management practices [9]. Accurate stem volume information is a primary input for fundamental forest inventories that help in economic forecasting, decision-making, and the sustainable planning of timber resources, thus it is indispensable for forest managers [10]. However, significant limitations on stem volume estimations include the lack of an accurate objective and an efficient methodological approach. Timber management decisions are directly related to revenues, which are closely tied to the economic evaluation of management activities, and thus accurate data on forest resources is required [11]. Traditionally, field-based methods of data collection have been employed for supporting the construction and update of forest inventories, but these methods are time-intensive and laborious. 

Stem circumference is usually defined by a circle. Clusters of points from 3D point clouds can be isolated, extracted, and estimated using different types of circle-fitting algorithms. Several methods exist for the estimation of stem diameter, and they can be mainly divided into two major categories: algebraic and geometric. The most common geometric methods include Levenberg-Marquardt and Gauss-Newton algorithms; other geometric approaches to approximate the diameter of stem sections include using cylindrical shapes [12,13], skeletonization [14,15], polygons [16], and Hough transformation [17,18]. Most algebraic methods, such as approaches by Kasa [19], Pratt [20], and Taubin [21], are usually faster and non-iterative compared to geometric methods. Pueschel et al. [22] studied several circle-fitting methods to consider the importance of outlier removals before applying any circle-fitting approach. In another study, Koreň et al. [23] used several circle-fitting methods using TLS data to compare the accuracies of diameter at breast height (1.3 m; DBH). Their study suggested that the optimal circle method based on the least square algorithm proved to be the most accurate circle-fitting method. 

The accuracy of DBH from TLS-based point clouds depends on several factors, including the scanning mode, the number of scans, scanner position, scanner settings (e.g., point density), and methods of data processing [24,25]. Also, to ensure high-quality TLS data, suitable environmental conditions (e.g., less windy) and forest conditions (e.g., occlusion effects) are required to ensure that stem-level characteristics are distinguishable by the TLS. For scenarios with multiple scans, point clouds are co-registered and combined with the help of matching. 

Hierarchical cluster analysis (HCA), whereby the number of clusters is not established a priori, hierarchically groups datasets using an unsupervised method for nested clustering. It is a robust method for finding homogenous groups based on their measured characteristics [26], and unlike factor analysis, HCA makes no distinction between independent and dependent variables.

The determination of ground surface is a semantic parameter that can significantly affect the extraction and estimation accuracy of tree height and stem diameter. For example, Maas et al. [27] used a digital terrain model (DTM) to distinguish ground surface points from other points based on the density allocation along the z-axis. A case from Guelph, Canada illustrated the performance of several ground-point classification algorithms using different features of interest (e.g., low vegetation, high vegetation). Interestingly, the cloth simulation filter (CSF) algorithm had the highest accuracy for high vegetation (forest type), producing a kappa of 0.844 [28]. In another study, Corte et al. [29] classified ground and off-ground points using the progressive TIN densification filtering technique available in LAStools for generating a DTM and digital surface model (DSM); they used a resolution of 0.5 m to extract total tree height. The use of canopy height models (CHMs) to identify treetops for the extraction of total tree height has been thoroughly studied [30,31,32,33,34,35].

Many studies have shown the practical advantages of TLS using different scaling approaches for estimating several stem parameters, such as height [36,37,38,39], crown width [37], stem diameter [37,38,39], and tree species recognition [39,40]. Other studies have been conducted for deriving plot BA [39,41], crown volume [42,43], leaf area index [44], and biomass [45]. However, TLS studies to estimate stem diameter are of great importance in forestry for assessing volumetric dimensions. Pueschel et al. [22] used a TLS to estimate the number of structural vegetation parameters by assessing the effects of scan mode and circle fitting on the extraction of stem diameter and volume. Their results showed that stem volume exhibited large variability with deviations from the reference volumes ranging from −34% to 44%. In another study, Astrup et al. [46] explored how volume estimates of individual trees from a harvester, standard volume functions, and TLS compared with standard inventory estimates. They found that individual tree volumes from TLS could be estimated with high precision and accuracy; Spearman correlation coefficients ranged between 0.77 and 0.97. In a recent study, Mayamanikandan et al. [47] assessed tree height and DBH to evaluate tree volume. Their results showed that TLS-based variables, such as DBH (R^2^ = 0.995), height (R^2^ = 0.998), and volume (R^2^ = 0.958) were in good accordance with the field measurements.

The main objectives of this study were to: (i) test the performance of TX8 survey-grade TLS for the construction of detailed 3D point clouds for extracting total tree height and DBH; we tested two height estimation methods in combination with a computationally robust algebraic circle-fitting algorithm through HCA; and (ii) assess the accuracy of these attributes for evaluating total stem volume by comparing them with the reference measurements, potentially allowing advancements in precision forestry.

## 2. Materials and Methods

### 2.1. Characterization of the Study Area

**Figure 1:**
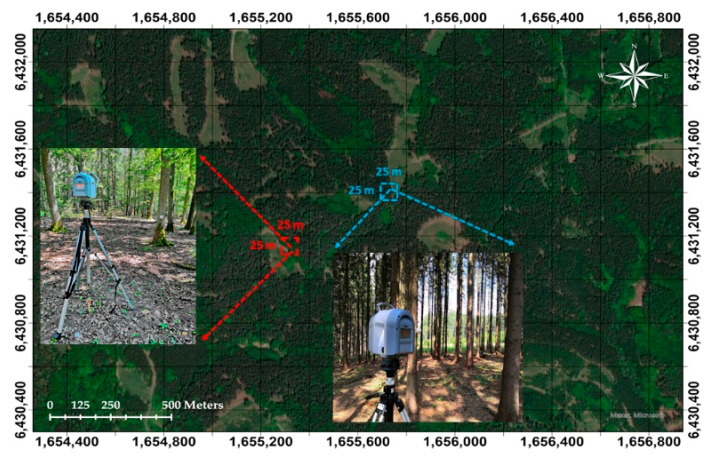
The geographic location of the study plots (plot 1 with red color and plot 2 with blue color). We used the coordinate system WGS 1984 Web Mercator (Auxiliary Sphere).

The study site is in the School Forest Enterprise of the Czech University of Life Sciences (ČZU Prague) in Kostelec nad Černými lesy, about 35 km southeast of Prague near the village of Oplany (Figure 1). The site contains two experimental plots that are each 625 m^2^ (25 × 25 m^2^). Plot 1 extends geographically from 49°54′45.15″ N; 14°52′11.44″ E to 49°54′44.98″ N; 14°52′12.75″ E, and plot 2 from 49°54′50.19″ N; 14°52′23.61” E to 49°54′50.12″ N; 14°52′24.98″ E. In terms of species composition, plot 1 is dominated by European oak (*Quercus robur*), and plot 2 by Norway spruce (*Picea abies* (L.) H. Krast.). The extended area is mainly characterized by managed, even-aged forests of approximately 50 to 60 years of age. The terrain profile in the area is slightly inclined, with an altitude of about 420 m a.s.l., mean annual temperature of 7.5 °C, and mean annual precipitation 600 mm.

### 2.2. Reference Measurements

Trimble M3 total station (Trimble Inc. Sunnyvale, CA, USA, 1978) was used to measure the tree positions and helped to verify the exact locations of the trees from TLS (detected trees). At 100 m the error range is ±23 mm. We measured 27 trees in plot 1 and 48 trees in plot 2.

Total tree heights were determined with a Haglöf Laser Geo (distance accuracy 40 mm), and DBH was determined using a Haglöf DP II (Haglöf Sweden AB, Långsele, Sweden, 2002) computer caliper with millimeter accuracy. Two diameters from perpendicular directions were measured and the DBH was then determined as the average value of these two measurements.

### 2.3. TLS Data Collection and Pre-Processing

The laser data were obtained using the Trimble TX8 scanning system (Trimble Inc., Sunnyvale, CA, USA.). The necessary technical specifications of the TLS device are shown in Table 1. The integrated high dynamic range (HDR) camera offered two different modes for image acquisition, standard and HDR. We used the standard mode for the colorization of the point clouds. To ensure an optimal degree of overlap between the scanning positions, we used the multi-scan approach with a total of seven scans. The first scan was placed at the center of each plot and the rest were spaced out along the periphery. In addition to the scanning parameters, fixed exposure was disabled and the third level scan density was used. Each scan took approximately 12 min; with a point spacing of 5.7 mm for a 30-m distance from the scanner position, they produced 555 million points for a single scan [48]. To calibrate the laser scanner, the field instant method was used, and to ensure better performance for the scan registration process, the laser scanner reference sphere set was used. In parallel with the scans, marked wooden sticks were placed on the ground at each scanning station, which allowed us to locate the scanning positions using the Trimble M3 total station. The error of the Trimble M3 total station was 2 mm along the horizontal plane.

The weather conditions were favorable for scanning on both plots for 29 June 2019, between 11:00–15:00 CET (plot 1) and 26 August 2019 between 12:00–16:00 CET (plot 2). The co-registration of the point clouds was conducted in RealWorks software (Trimble Inc., Sunnyvale, CA, USA) with an accuracy of 1.42 mm. To ensure an adequate number of points mainly in the upper portions of the trees, near the treetops, the point clouds were sampled at 0.01 m, thus producing a point density of approximately 132,000 p/m^2^.

### 2.4. Normalization Height

After the pre-processing phase, the point clouds were extracted and imported in CloudCompare software V.2.10. (Zephyrus, Paris, France, 2011), where the CSF algorithm [49] was applied to separate and extract the ground from non-ground points. In the general parameter setting tab of the surface base filter, the relief terrain option was chosen due to the slightly inclined plane in both plots.

**Figure 2:**
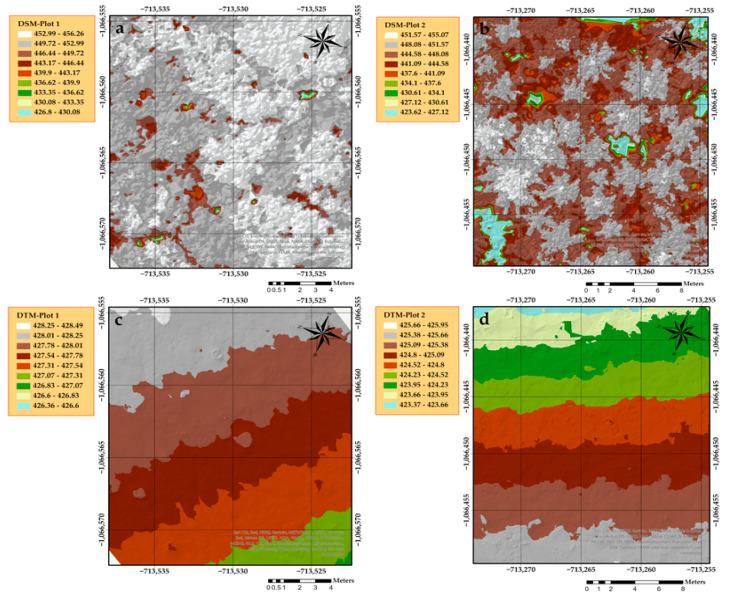
The extracted digital surface models DSMs (**a**,**b**) and digital terrain models DTMs (**c**,**d**) in both plots using LAStools in ArcGIS.

For calibration, cloth resolution was set to 1.1; as for maximum iterations we used the default option (500 iterations). It was necessary to classify all the points into ground and off-ground layers to eliminate the differences in tree height caused by differences in elevation, which we did by computing the distances between ground (reference points) and non-ground points (compared points); the classification threshold was set to 0.1 (the unit was the same as the unit of point clouds). Ground and off-ground points were then extracted as las files and imported into ArcGIS desktop V.10.6.1 (ESRI Inc.; Redlands, CA, USA) to create the DSM and DTM (Figure 2) with an accuracy of 0.01 × 0.01 m^2^ cell size. The process of normalization height was continued using the LAStools (rapidlasso GmbH, Gilching, Germany) toolbox in ArcGIS.

### 2.5. Tree Height Estimation from H_TTD_ and H_TSP_

By overlaying the normalized CHMs (nCHMs) and the extracted tree stump positions from the TLS using the centers of stem cross-sections at stump height (30 cm) [50], we were able to both validate tree positions and extract the total tree heights using the Feature to Point tool in ArcGIS. For extracting total tree heights, we considered a hypothetical vertical vector along the *z*-axis passing from the centers of each stem cross-section at stump height up to the treetop, taking into account only the closest points to that central point’s values. For simplicity, we referred to this height as H_TSP_.

For the second height estimation method, the local maxima algorithm [51] was applied on the nCHMs for treetop detection, as described in [33], and thus we could estimate the total tree height; we referred to this height as H_TTD_. The setting parameters for the local maxima were determined using the morphological filtering tool in focal statistics. During the processing, we tested several sizes of circular filters to determine what would be the ideal kernel window size for optimal treetop detection in each plot. We concluded that the ideal kernel window size was undeniably 1 m for plot 1 because it resulted in at least one treetop per tree, thus ensuring (i) the minimum number of missing trees, and (ii) the lowest amount of multi-treetop stems. A kernel window size of 2 m was applied in plot 2; here the decision for optimal kernel window size was more straightforward because the application of local maxima on coniferous trees can successfully detect a single treetop per tree [33]. Thus, in plot 2 only max values were considered in the estimations from H_TTD._ Due to the multi-treetop nature of the broadleaves, we considered not only the $H_{\mathrm{TTD}}\mathrm{MAX}$, but the $H_{\mathrm{TTD}}\mathrm{MEAN}$ values of detected treetops in each tree. We assumed that the mean values of detected treetops will smooth the estimated heights in plot 1 and decrease the modeling error. To match the pixel values from nCHMs and the focal statistic result we used Equation (1):

(1) Con(‘nCHM’==‘focal statistics result’1),

The conditional function (Con; Equation (1)) performs an if/else evaluation on each input cell of the input raster and assigns a binary value of 0 (for non-data) or 1 (for data value). The assigned value was the point at which the nCHM value equaled the focal statistic output. Total tree height from H_TTD_ and H_TSP_, was estimated based on the nCHM.

### 2.6. Estimation of Diameter at Breast Height

**Figure 3:**
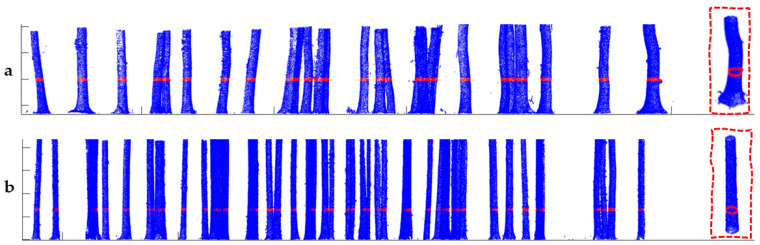
Illustration of segmented stem profiles including an example of the distribution of points along the stem circumference at DBH (inside the red rectangular outlines) in plot 1 (**a**) and plot 2 (**b**). Points with red color represent the selection of all points within the buffer zone centered at DBH.

The normalized points were used as input for the estimation of DBH. A buffer zone (Figure 3) was applied to select all points between 1.25 and 1.35 m above ground level using the Net Framework in Visual Studio Enterprise 2015 V.14.0.00 (Microsoft©, Redmond, Washington, DC, USA, 1975). The average number of selected points used to estimate tree diameter for each tree was approximately 95 in plot 1 and 86 in plot 2. The points were then extracted as txt files for further processing.

We then estimated the stem radius in the produced stem cross-sections using the HCA method [52]. This method enabled the creation of several clusters (one for each cross-section) based on the total number of trees present in each plot. First, we calculated the Euclidean distances between the (x, y) points using the *pdist* function (Equation (2)):

(2) $$d\left(x,\;y\right)=\;\sqrt{(x_{1}-y_{1}){\;}^{2}+\;{\left(x_{2}-{\;y}_{2}\right)}^{2}}$$

where (x, y) are two points with coordinates x = ($x_{1},{\;x}_{2}$) and y =$\;\left(y_{1},{\;y}_{2}\right)$ in two-dimensional space.

Once the proximity between points has been computed, we determined how the stem cross-section points should be grouped into clusters using the *linkage* function. Ward’s minimum variance method in the *linkage* function was used to obtain the calculated distances and link pairs of (x, y) points into binary clusters (Equation (3)):

(3) $$\;d\left(r,s\right)=\sqrt{\frac{2n_{r}n_{s}}{\left(n_{r}+n_{s}\right)}}\times \|{\;\bar{x}}_{r}-{\bar{x}}_{s}\|{}_{2}$$

where $\|{\|}_{2}$ is the Euclidean distance, ${\;\bar{x}}_{r}$ and ${\bar{x}}_{s}$ are the centroids of clusters (r) and (s), respectively, and $n_{r}$ and $n_{s}$ refers to the number of elements in clusters (r) and (s), respectively.

The *cluster* function was then used to specify arbitrary clusters to partition data into the desired number of clusters based on the total number of trees in each plot. We used the algorithm proposed by Bucher [53], which is a modified version of the [19] circle fit method (Equation (4)), based on the least-squares. The *circfit* function was used to fit a circle to a set of measured points.

(4)  [xc, yc,R,a~]=circfit(x, y)

where xc,yc are the returned centers for each stem cross-section, R is the returned estimated radius, and the fourth parameter, a, is an optional coefficient describing the general circle form (Equation (5)).

(5) $$x^{2}+y^{2}+a1\times x+a2\times y+a3=0$$

The constructed *circfit* function was iterated to ensure that simultaneously all stem cross-section points are fitted, their respective radii are estimated, and all the extracted information (e.g., number of trees in each plot) will be successfully stored in cell arrays. The entire processing phase was conducted in Matlab R2017b professional edition (MathWorks©, Inc., Natick, MA, USA) using the Statistics and Machine Learning Toolbox™.

### 2.7. Total Stem Volume Estimation

Equation (6) was used to calculate total stem volume using DBH, total tree height, and form factor (F) as predictors for both measured and estimated values. For the estimated heights, the height values from both methods (H_TTD_ and H_TSP_) were considered for the estimation of total stem volume. For stem diameter, the radius of each stem was converted to DBH.

(6) $$V_{\mathrm{oak}/\mathrm{spruce}}=\;\mathrm{DBH}\times H\times F\;$$

where V_oak/spruce_ refers to the total stem volume and the respective species, H is total tree height, and F is the stem form factor. Based on the allometric equations by Petráš and Pajtík [54], F = 0.38 for European oak and F = 0.42 for Norway spruce.

DBH was then converted to area using Equation (7):

(7) $$g_{\mathrm{stem}}=\pi \;\times \;\frac{{\left(\mathrm{DBH}\right)}^{2}}{4}$$

where g_stem_ refers to the BA and π is a mathematical constant.

### 2.8. Accuracy Assessment

Once tree heights and diameters were extracted, the accuracy of the proposed method was then evaluated. The estimated heights, H_TTD_ and H_TSP_, were compared with the reference measurements to test if there were any statistically significant differences between them. For the measured versus estimated diameters, we used a paired *t*-test with a 0.05 significance level.

Finally, an analysis of variance (ANOVA) was used to test the degree of variability within the regression models (measured versus estimated total stem volumes). To evaluate the accuracy of the volume models, we used the following evaluation metrics:

(8) $$R^{2}=1-\frac{\sum^{}{\left(y_{i}\;-\hat{y}\right)}^{2}}{\sum^{}{\left(y_{i}\;-\bar{y}\right)}^{2}\;}$$

where $\hat{y}$ is the predicted value of y and $\bar{y}$ is the average value of y.

(9) $$R_{\mathrm{adj}}^{2}=1-\left[\frac{\left(1-R^{2}\right)\left(n-1\right)}{n-k-1}\right]$$

where R^2^ refers to the sample R-square, k is the number of predictors, and n is the total sample size.

(10) $$\mathrm{SE}=\frac{\sigma }{\sqrt{n}}$$

where σ is the standard deviation and $\sqrt{n}$ is the number of samples.

(11) $$\mathrm{RMSE}=\sqrt{\frac{\sum_{i=1}^{n}{\left(\mathrm{Vol}.{\mathrm{Estimated}}_{i}-\mathrm{Vol}.{\mathrm{Measured}}_{i}\right)}^{2}}{n}}$$

where $\mathrm{Vol}.{\mathrm{Estimated}}_{i}$ is the predicted volume, $\mathrm{Vol}.{\mathrm{Measured}}_{i}$ is the observed volume, and n is the number of observations.

(12) $$\mathrm{RMSE}\%=\frac{\mathrm{RMSE}}{\bar{x}}\times 100$$

where RMSE is the root mean square error and $\bar{x}$ is the average value.

(13) $$\mathrm{Bias}=\;\frac{1}{n}\;\sum x_{i}-{\bar{x}}_{j}$$

(14) $$\mathrm{Bias}\%=\;\frac{\mathrm{Bias}}{{\bar{x}}_{j}}\times 10$$

where n is the number of samples, $x_{i}$ the observed value, and ${\bar{x}}_{j}$ is the average of estimated values.

(15) $$\mathrm{MAE}=\frac{\sum_{i=1}^{n}\left|p_{i}-x_{i}\right|}{n}$$

where $p_{i}$ is the predicted value, $x_{i}$ the observed value, and n is the number of observations.

**Figure 4:**
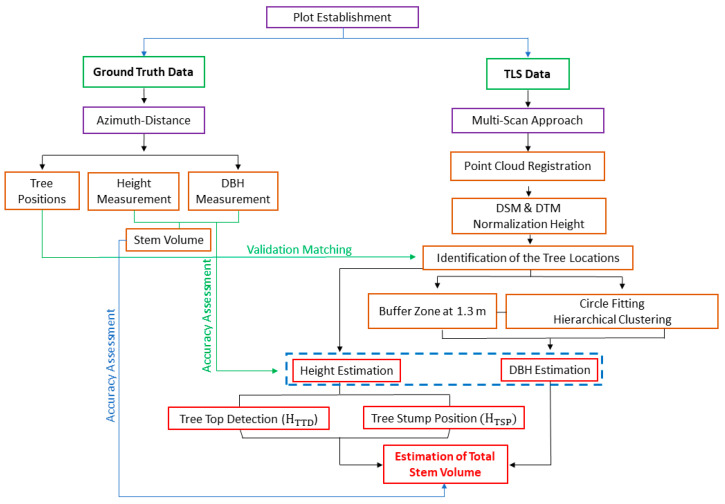
Displays the workflow for estimating total stem volumes.

The entire statistical analysis was conducted in Matlab R2017b professional edition (MathWorks©, Inc., Natick, MA, USA). An overview of the workflow can be seen in Figure 4.

## 3. Results and Discussion

### 3.1. Modeling Tree Height

**Figure 5:**
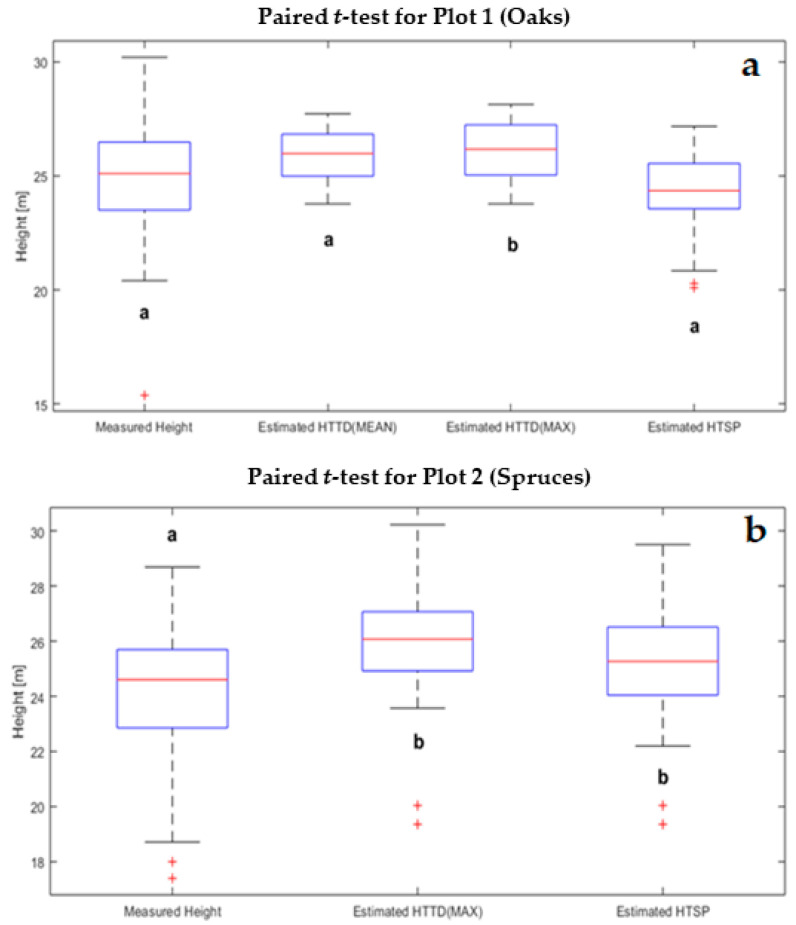
Box plots for measured and estimated tree heights for plot 1 (**a**) and plot 2 (**b**). The letters (a, b) above and below box plots indicate if there were significant differences between the groups at a 0.05 significance level.

Total tree height from TLS was validated by comparing it to reference measurements. In plot 1 we observed a significant difference between measured tree heights and $H_{\mathrm{TTD}}\mathrm{MAX};$ there were no significant differences between the other estimates, $H_{\mathrm{TTD}}\mathrm{MEAN}$ and H_TSP_ (Figure 5). The average measured height was 24.86 m; H_TSP_ was estimated to be 24.14 m, $H_{\mathrm{TTD}}\mathrm{MEAN}$ was 25.85 m, and for $H_{\mathrm{TTD}}\mathrm{MAX}$ 26.08 m. In plot 2, both H_TSP_ and $H_{\mathrm{TTD}}\mathrm{MAX}$ were significantly different than the measured height values (Figure 5). The average of measured heights was 24.19 m; H_TSP_ was 25.11 m and $H_{\mathrm{TTD}}\mathrm{MAX}$ 25.96 m.

Both tree height estimation methods, H_TTD_ and H_TSP_, overestimated reference heights on both plots, except in the case of H_TSP_ in plot 1 that suggested a slight underestimation of height (Figure 5). In plot 1, relative to the measured heights, the $H_{\mathrm{TTD}}\mathrm{MEAN}$ outperformed $H_{\mathrm{TTD}}\mathrm{MAX}$, with a height difference of 0.99 m compared to 1.22 m, respectively, and H_TSP_ outperformed both with a height difference of only 0.72 m. In plot 2, H_TSP_ yielded estimates closer to the measured heights, 0.92 m, compared to the extracted height from $H_{\mathrm{TTD}}\mathrm{MAX}$, 1.77 m.

**Figure 6:**
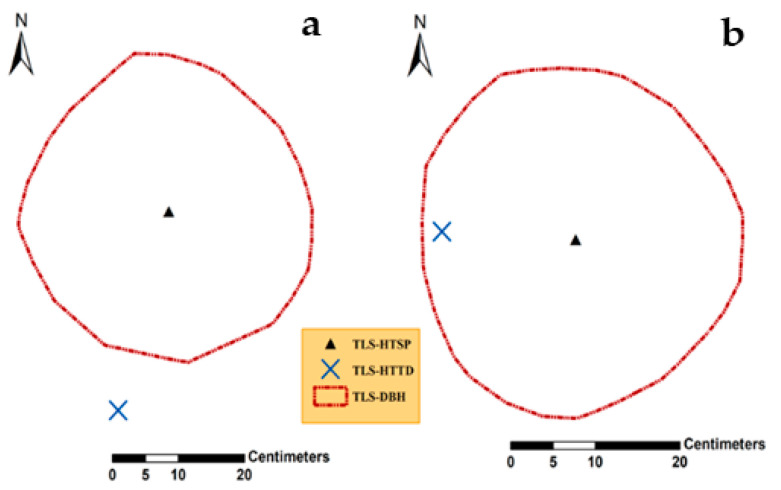
Illustrates the degree of stem deflection along height in two different spruce stems using nadir view in ArcGIS. The deflection is more visible in the case of H_TTD_ for cross-section (**a**) compared to cross-section (**b**), while for H_TSP_ that difference is negligible in both cases.

Underestimation of tree height by H_TSP_ in plot 1 was attributed to the stem morphological characteristics of broadleaves. In plot 2, overestimation of tree height by both methods, H_TSP_ and $H_{\mathrm{TTD}}\mathrm{MAX}$, was likely caused because coniferous trees tend to have more cylindrical shapes, hence the locations of treetops were perfectly aligned with the centers of all stem cross-sections in stump height (Figure 6). However, H_TSP_ in plot 2 underestimated tree height compared to H_TTD,_ as expected. Except for H_TSP_ in plot 1, our result differed from the results from [55].

Also, the moderate crown closure in both plots allowed researchers to catch higher stem portions in most trees. In general, the vertical angular step-width of the TX8 laser scanning is relatively small compared to less expensive scanners, therefore the point density is higher in the vertical paths. Overall, except for H_TSP_ in plot 1, our findings are consistent with the results from [56,57].

Broadleaved and coniferous trees have very different growth morphologies. Coniferous trees have vertical, straight-lined stems, and their crowns tend to be clustered. In contrast, broadleaves tend to have a more curved stem and wider coverage of the crown. Consequently, coniferous forest canopies tend to have more open space, thus relatively accurate tree heights can be obtained within a certain range of the scanner when the stations are properly arranged [58]. Several studies have demonstrated that due to the mutual occlusion between canopies in dense coniferous and broadleaves forests, TLS cannot obtain an efficient amount of point cloud information at the top of the canopy, which results in an underestimation of tree heights [58,59,60]. Our results demonstrated that this issue can be partially addressed using mean values for broadleaves and maximum values for conifers in combination with the small vertical angular step-width of the TX8 laser scanner, which produced higher point densities in the vertical directions. The proposed methodology can provide reliable information for tree height estimations up to 30 m from the ground.

**Figure 7:**
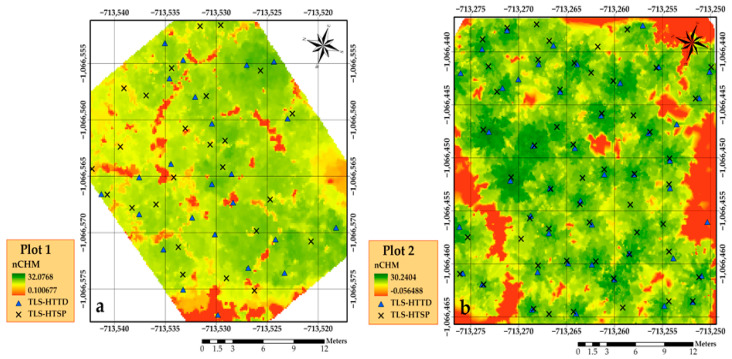
Normalized canopy height models (nCHMs) and the locations of estimated tree heights from both methods (H_TTD_ and H_TSP_) in plot 1 (**a**) and plot 2 (**b**). All trees were detected from TLS H_TSP_ in both plots.

Figure 7 displays the produced nCHMs with the locations of tree positions from TLS overlayed with the positions from the local maxima. We can also see the effect of multi-treetop detection by the local maxima (H_TTD_) for plot 1 compared to plot 2, where a single treetop corresponded to a single tree.

### 3.2. Modeling Diameter at Breast Height

**Figure 8:**
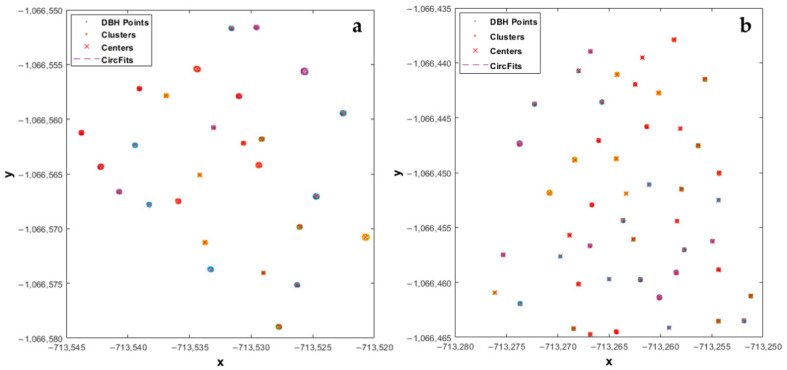
Tree positions and the extracted stem cross-sections at DBH, including the application of the *circfit* algorithm for the diameter estimation based on hierarchical clustering analysis (HCA) in plot 1 (**a**) and plot 2 (**b**). Every cluster is depicted with a different color.

**Figure 9:**
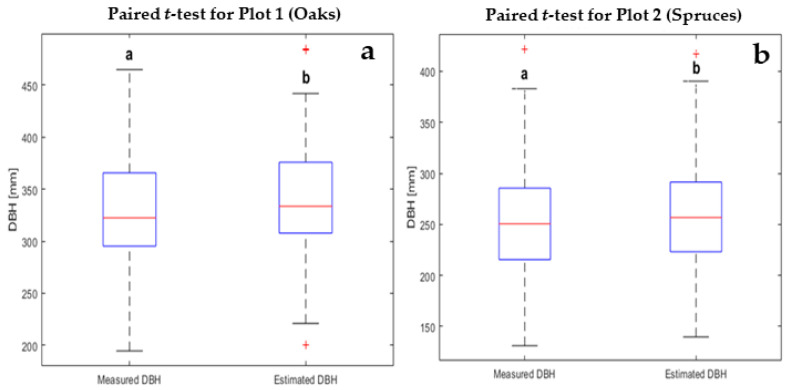
Statistical comparison between measured and estimated diameters at breast height (DBH) in plot 1 (**a**) and plot 2 (**b**). The letters (a, b) above the box plots indicate if there were significant differences between the groups at a 0.05 significance level.

Using the *circfit* algorithm through the application of HCA, compact and well-separated clusters were created for all the trees in both plots (Figure 8). A cross-section of each plot shows the well-rounded scanned stems, regardless of the tree size. The proposed method presented enhanced detection capability and high accuracy (Figure 9). Furthermore, the algorithm to extract tree DBH minimized the sum of squared radial deviations, thus producing smaller errors and lower bias.

We suggest these results can be attributed to the point cloud density setting of 1 cm, the application of the multi-scan approach, which covered all the trees from several angles, and the device’s capacity to adjust to a small angular step-width in the horizontal direction. 

A statistical comparison between the measured and estimated diameters showed a significant difference (*p*-value < 0.05). The estimated DBHs were consistent with the reference measurements, but DBHs derived from TLS were slightly overestimated [61] in both plots (Figure 9). In plot 1, the average of the measured diameters was 329.46 mm compared to the estimated value of 339.72 mm. Similarly, in plot 2 the average value for the measured diameters was 257.17 mm compared to an average estimated DBH of 262.64 mm. The difference between estimated and measured diameters was 10.26 mm and 5.47 mm in plots 1 and 2, respectively (Figure 9), a difference of 4.79 mm between the estimated diameters in both plots. Our findings were consistent with the results of [22,56,62,63,64].

Our findings showed similar accuracy in the estimation of DBH in both plots. Although broadleaved (plot 1) and conifer (plot 2) trees have different morphological characteristics, their stems are nearly circular and mostly cylindrical close to the ground. Slight overestimates in both plots can be associated with the TLS data (e.g., registration error, noise). On the other hand, broadleaved tree species present different bark textures and structures (e.g., deep ridges) that could affect the diameter measurements more compared to coniferous tree species.

### 3.3. Modeling Total Stem Volume

**Figure 10:**
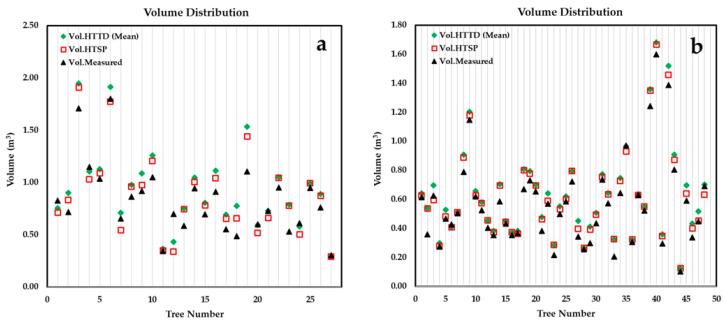
Graphical relationship between total stem volume from three different methods in plot 1 (**a**) and plot 2 (**b**).

As evident in Table 2 and Figure 10, an identical pattern between the estimated and measured total stem volumes ($\mathrm{Vol}._{H_{\mathrm{TTD}}}>\mathrm{Vol}._{H_{\mathrm{TSP}}}>\mathrm{Vol}._{\mathrm{Measured}}$) was observed for both plots In plot 1, the estimated volume from H_TSP_ outperformed the H_TTD_ method, a difference of 1.228 m^3^; the H_TTD_ estimated volume was comparable with a difference of 2.732 m^3^. The difference between the estimated volumes from H_TTD_ and H_TSP_ was just 1.504 m^3^.

Plot 2 also exhibited differences between measured and the two estimated volumes derived from H_TTD_ and H_TSP._ Although the differences between measured and estimated volumes were higher for plot 2, the difference between estimated volumes from H_TTD_ and H_TSP_ was lower (0.965 m^3^). The difference between estimated volumes from the H_TTD_ and H_TSP_ methods and the measured volumes was 2.971 m^3^ and 2.006 m^3^, respectively.

Overall, our results revealed an overestimation of total stem volume compared to the measured values for both height estimation methods in both plots. Our findings are in line with the results of previous studies [3,65,66,67]. 

The differences between estimated and measured total stem volumes were statistically significant in both plots with tested *p*-values < 0.05 (Table 3 and Table 4). The coefficient of determination between the measured and estimated volumes from H_TTD_ and H_TSP_ was very high in plot 2, R^2^ = 0.98, whereas in plot 1 the relationships were weaker with R^2^ = 0.89 for H_TTD_ and R^2^ = 0.87 for H_TSP_. As a measure of the standard deviation of the average within the datasets, the standard error was lower in plot 2 (0.043) for both height estimation methods. For plot 1 the standard error was higher, yet insignificant, with 0.134 for H_TTD_ and 0.143 for H_TSP_. These numbers indicate a small spread of values in both cases, and, therefore, high dataset accuracy (Table 3 and Table 4).

The validation metrics of total stem volume estimation for both plots are displayed in Table 5. In plot 1, the volume estimation from H_TSP_ performed better compared to the H_TTD_ with an RMSE = 0.14, RMSE% = 17.51, Bias = −0.338, Bias% = −38.63, and MAE = 0.119. The other method presented lesser, yet comparable performance with an RMSE = 0.17, RMSE% = 20.01, Bias = −0.307, Bias% = −32.98, and MAE = 0.132. In plot 2, the volume estimation from H_TSP_ yielded superior performance compared to the H_TTD_ with an RMSE = 0.06, RMSE% = 10.84, Bias = 0.042, Bias% = 6.85, and MAE = 0.048. Volume estimates using the H_TTD_ method presented a lesser, yet comparable performance with an RMSE = 0.08, RMSE% = 13.26, Bias = 0.062, Bias% = 9.82, and MAE = 0.063 (Table 5).

## 4. Conclusions

The main merit of this study was to investigate the technical capabilities of the Trimble TX8 laser scanning system to accurately estimate the total stem volume of individual trees. We demonstrated the use of two different height estimation methods (H_TTD_ and H_TSP_) applied to detailed nCHMs to extract total tree height from multi- and single-treetop trees using different descriptive statistics. It is worth mentioning that a direct comparison between different studies is a challenging task because there is high uncertainty in the way field measurements are conducted, as well as the diverse forest conditions from each study. Under these circumstances, how accurate and reliable are field measurements to be used as reference data?

The conifer trees (plot 2) typically have just a single treetop that corresponded to one tree; however, for the multi-treetop broadleaf trees (plot 1), we assumed that mean tree height would be more meaningful. A comparison of the measured and estimated heights revealed that the mean treetop values performed better for broadleaves, and, as we had assumed, it also resulted in higher accuracy. Underestimation of tree height using H_TSP_ in plot 1 was attributed to the stem morphological characteristics of broadleaves. In contrast, overestimation of tree height in plot 2 was related to the cylindrical shapes of the coniferous trees, hence the locations of treetops were perfectly aligned with the centers of all stem cross-sections at stump height. Also, the proposed method in combination with the TX8 TLS proved to be reliable for tree height estimation up to 30 m. We attributed this to the small vertical angular step-width of this particular laser scanner, which provided for higher point densities in the vertical direction.

According to the results from *circfit* and HCA for the extraction of stem DBH, well-separated clusters were created, thus suggesting the robustness of the proposed method (Figure 8). The statistical analysis comparing extracted and reference values verifies the accuracy of the model to extract tree DBH regardless of the tree size.

As demonstrated by the coefficient of determination, there was high agreement between measured and estimated total stem volumes from both height methods (H_TTD_ and H_TSP_) in both plots. Even though better accuracy was found between measured and estimated total stem volumes from H_TSP_ based on RMSE% (Table 5), the differences in volume from the two height estimation methods in both plots were rather low; for example, for plot 2 the accuracy reached almost 90%.

In this study, differences between measured and estimated attributes were small. Conclusively, the proposed method is feasible for semi-automatic stem volumetric modeling of small-medium scale studies and can be used not only for practical (e.g., expeditious construction of single-entry volumetric tables), but for scientific purposes too.

## Figures and Tables

**Table 1:** Main performance indicators of Trimble TX8 terrestrial laser scanning device.

**Range Measurement**	
Maximum Distance Range	120 m on most surfaces
Range Systematic Error	<2 mm
Laser Wavelength	1.5 µm, invisible
Laser Beam Diameter	6–10–34 mm @ 10–30–100 m
**Scanning**	
Scanning Field-of-View	360° × 317°
Scanning Speed	1 million pts/sec
Angular Accuracy	80 µrad

**Table 2:** Shows the extracted total stem volumes based on two different height estimation methods as well as the measured total stem volumes.

Plot ID	Parameter	Estimated Volume $(H_{TTD})\;*$	$\mathrm{Estimated}\;\mathrm{Volume}\;(H_{TSP})$	Measured Volume
1	Total Stem Volume in (m^3^)	24.903	23.399	22.171
2		30.261	29.296	27.290

* $H_{\mathrm{TTD}}$: Plot ID 1 consider the mean and plot ID 2 the max height values.

**Table 3:** Analysis of variance (ANOVA) and regression statistics showing the relation between measured and estimated volumes using estimated height derived from the local maxima (H_TTD_).

Plot ID		R-Square	Adjusted R-Square	Standard Error	df	SS *	MS *	F *	Prob > F *
	Regression	0.89	0.89	0.134	1	3.67	3.67	203.226	0.00
1	Residual	-	-	-	25	0.45	0.02	-	-
	Total	-	-	-	26	4.12	-	-	-
	Regression	0.98	0.98	0.043	1	4.48	4.48	2417.746	0.00
2	Residual	-	-	-	46	0.09	0.00	-	-
	Total	-	-	-	47	4.57	-	-	-

* SS: sum of squares; MS: the mean squares for each source; F: F-value Prob > F: the returned *p*-value.

**Table 4:** Analysis of variance (ANOVA) and regression statistics showing the relation of measured and estimated volumes using estimated height derived from tree stump positions (H_TSP_).

Plot ID		R-Square	Adjusted R-Square	Standard Error	df	SS *	MS *	F *	Prob > F *
	Regression	0.87	0.87	0.143	1	3.46	3.46	169.992	0.00
1	Residual	-	-	-	25	0.51	0.02	-	-
	Total	-	-	-	26	3.97	-	-	-
	Regression	0.98	0.98	0.046	1	4.32	4.32	2027.982	0.00
2	Residual	-	-	-	46	0.10	0.00	-	-
	Total	-	-	-	47	4.42	-	-	-

* SS: sum of squares; MS: the mean squares for each source; F: F-value Prob > F: the returned *p*-value.

**Table 5:** Evaluation metrics for total stem volume accuracy.

Plot ID	Volume in (m^3^)	RMSE *	RMSE% *	Bias	Bias%	MAE *
1	H_TTD_	0.17	20.01	−0.307	−32.98	0.132
	H_TSP_	0.14	17.51	−0.338	−38.63	0.119
2	H_TTD_	0.08	13.26	0.062	9.82	0.063
	H_TSP_	0.06	10.84	0.042	6.85	0.048

* RMSE: root mean square error; * RMSE%: root mean square error percentage; * MAE: mean absolute error.

## Data Availability

Restrictions apply to the availability of these data. Data was obtained from [Department of Forest Management; Faculty of Forestry and Wood Sciences of the Czech University of Life Sciences Prague] and are available [from the authors] with the permission of [Czech University of Life Sciences Prague].
